# Sex differences in college students' knowledge of concussion and concussion education sources

**DOI:** 10.2217/cnc-2023-0001

**Published:** 2023-05-03

**Authors:** Stephen J Heck, Amanda Acord-Vira, Diana R Davis

**Affiliations:** 1 Social & Behavioral Sciences, School of Public Health, West Virginia University, Morgantown, WV 26506, USA; 2 Division of Occupational Therapy, West Virginia University, Morgantown, WV 26506, USA

**Keywords:** college students, concussion, educational needs, prevention, traumatic brain injury

## Abstract

**Aim:**

To understand sex differences and sources of concussion education for college students. The literature for college students primarily focuses on sports concussions and general knowledge. Understanding how non-students-athletes learn is critical to developing interventions to improve concussion knowledge.

**Participants:**

A random sample of 208 students from four-year institutions.

**Methods:**

A 22-question online survey explored postsecondary students' current knowledge and education regarding concussions.

**Results:**

Findings indicated that sex differences emerged with concussion knowledge and sources of concussion knowledge (e.g., leaflets, pamphlets, parents, and television). The top choices for where they wanted to learn about concussions were health educators, health centers, and campus peer educators.

**Conclusion:**

This study provides an initial evaluation and implications for future research on providing concussion education.

Traumatic brain injuries (TBI) are a critical public health issue, and society must find ways to educate and understand how communities respond [1]. According to the CDC, in USA, approximately 2.8 million TBIs are reported annually [2]. TBIs are one of the key causes of death and disabilities (e.g., falls, motor vehicle accidents, being struck by an object, and assault) and contribute to around 30% of all injury-related deaths [2]. TBIs are disruptions to brain functions by either a shock, blow or bump to the head and range in severity from mild to severe [2]. A mild TBI includes concussion, and the terms are often interchangeable; subsequently, this paper will use the term concussion. A direct force to the head or body, which causes the brain to hit the skull, is the cause of a concussion [3]. People who sustain concussions can have severe and lasting difficulties [4]. Health issues can include memory problems, visual impairment, hearing impairment, and emotional/psychological changes; however, concussions do not always present with the same symptoms, creating challenges with proper identification [5].

Individuals ages 15–24 years have the second highest rate of TBI-related emergency department visits, the second highest rate for TBI-related hospitalization, and the third highest rate for TBI-related deaths [6]. Much of the literature for this age group focuses on sports-related concussions (SRC) [7–9], and concussion education is becoming a requirement at all levels of sports [10–13]; however, there are limited education programs or intervention studies conducted on the non-athletes.

Early concussion identification is essential to recovery, and a lack of education causes delays in concussion identification, treatment, and reporting [14]. Athletes can access sports medicine staff and education to aid in concussion identification and treatment [15]. Early identification provides the most health benefits with recovery and offers the best chance to return to activity [16]. When there is a lack of education, concussion identification and reporting delays can happen. In studies of athletes, delays in reporting can impact recovery time, academics, and sport participation [16]. Chinn and Porter found that even though student-athletes receive education, they may not know when they have a concussion [17]. Being in the moment of play, the athlete may not recognize it; their peers, coaches, parents, and athletic trainers need to know how to identify concussions. Similarly, with non-athletes, a concussed person may have difficulty identifying their trauma. It may require peers, family, colleagues, and bystanders to know concussion signs and symptoms.

## Sex differences

It is essential to look at the sex difference in concussions. Males are approximately 40% more likely to have a TBI [18], females are more likely to have brain injuries from assault or violence, and males with falls and vehicle accidents [19]. The brains of males and females respond differently at a cellular and molecular level and how the brain repairs and recovers, leading to worse outcomes in females than males [19].

Sex differences in concussion studies primarily focused on athletes' symptoms, risk factors, knowledge, and behaviors [20–23]. Marar and colleagues found that the overall concussion rate was higher in females than males in similar sports, and females have a higher proportion of recurrent concussions than males [21]. A systematic review of the effects of concussions on female athletes found that females have more prolonged symptoms and are more likely to report a concussion than males [24]. There are a variety of factors that influence different outcomes from a concussion for females. Research suggests there is a greater vulnerability for females to have a concussion than males due to neck strength [9,25–27], the neuroanatomy of the brain [9,25,28,29], and the role of the estrogen hormone [9,25,27,29]. Wallace and colleagues studied sex differences in knowledge of concussion symptoms and reporting behavior [22]. They found that females had higher knowledge of SRC and that males were 4 to 11 times more likely not to report a concussion. Concussion education needs to take sex differences into account and reduce the social stigma associated with this type of injury [22]. A better understanding of sex differences may lead to better identification, treatment, and outcomes [19].

## Concussion education

Research, state/federal legislation, and media have increased public awareness of the risk of a concussion [30]. According to Mrazik and colleagues, a wide range of media is used to educate athletes and others on concussions [31]. This includes print materials, handouts, pamphlets, posters, concussion cards, position statements from professional organizations, webinars, and websites. According to Carroll-Alfano, legislation in 43 states has mandated concussion education for high school student-athletes [32]. Through the CDC, the federal government offers HEADS UP, an online concussion training for youth sports [33]. With the National Collegiate Athletic Association (NCAA), there is a concussion education requirement for all athletes. In US collegiate sports, the primary educator is the athletic trainer; however, there is a desire for athletes to learn about concussions from their coach in addition to the athletic trainer and medical professionals [11]. While many resources and information on concussions are readily available, limited studies have evaluated their effectiveness [31]. There is a need for evidence-based reviews of these resources to ensure that these programs provide accurate information and best practices. Concussion education is needed to ensure that what athletes and non-athletes receive is adequate.

## General knowledge

A lack of knowledge, misconceptions, perceived susceptibility, perceived severity, and miscommunication can lead to harmful actions and stop people from seeking medical treatment for concussions [14]. According to Register-Mihalik and colleagues, education must be across multiple levels of society to prevent, identify, and manage concussions [12]. The current practice provides concussion education material to athletes, coaches, and families [10,34]. There have been studies on athletes and concussion education, but there are limited studies on non-athletes. Recognizing symptoms and treatment options is crucial to avoiding a concussion's negative and severe consequences [14].

The purpose of this study was to evaluate sex differences where participants learn and want to learn about concussions, rating of concussion knowledge, and overall concussion knowledge. This analysis has three framing questions: (1) what are the sources of concussion knowledge among college students? Does this differ by sex? (2) how do students want to learn about concussions? Does this vary by sex? and (3) Are there sex differences in concussion knowledge?

## Methods

### Procedure

This study used descriptive and convenience sampling to answer the framing questions. Before participant recruitment, the West Virginia University Office of Research Integrity & Compliance determined it was IRB exempt. Researchers randomly selected 500 institutions from the Carnegie Classification list of 4-year institutions. University staff at the student life departments were contacted through email and asked to distribute information regarding participation in this study to their student body. This email provided a survey link, study details, and a draft email for their students. In addition, multiple social networking platforms included basic information and a survey link as a recruitment strategy. The link provided to the student life departments and social media connected them to a web-based Qualtrics^™^. Following the information letter at the start of the assessment, students completed the anonymous survey after acknowledging that they met the inclusion criteria. The participants had to be 18 or older to complete the survey in English and currently enrolled in one credit hour at a college or university.

### Participants

The inclusion criteria for this study were for the person to be 18 or older, enrolled in postsecondary education, and able to read English. Based on self-reported college enrollment, 208 students who met the inclusion criteria from these selected universities completed the anonymous survey. The mean age for participants was 22.28 (SD = 5.81), ranging between 18 and 63. As the inclusion requirements for this study were only for the person to be 18 years or older and enrolled in postsecondary education, the mature students were not excluded from the analysis. As this study aimed to evaluate sex differences with where participants learn and want to learn about concussions, rating of concussion knowledge, and overall concussion knowledge, age was not a specific variable for this study. Table 1 provides the basic demographic characteristics of the sample. Sample demographics were 65.4% female, 30.8% male, 0.5% transgender male, and 0.5% genderqueer compared with the National Student Clearinghouse Research Center (NSCRC) term enrollment dataset for Fall 2022 with 58% female and 42% male and (at this time the NSCRC does not report on nonbinary students) [35]. The sample was predominately Caucasian at 84.1% compared with 47% from the NSCRC data. There was an even sampling across all groups with class designation, with seniors being the largest group at 31.7%.

**Table 1.** Demographic characteristics of the sample (n = 208).

Characteristics	n	%
Sex:		
– Male	64	30.8%
– Female	136	65.4%
– Transgender male	1	0.5%
– Genderqueer	1	0.5%
– Did not report	6	2.9%
Ethnicity:		
– Caucasian	175	84.1%
– Hispanic or Latino/a	4	1.9%
– Black or African–American	13	6.3%
– Native American	1	0.5%
– Asian/Pacific Islander	3	1.4%
– Other	7	3.4%
– Did not report	5	2.4%
Class designation:		
– Freshman	29	13.9%
– Sophomore	37	17.8%
– Junior	31	14.9%
– Senior	66	31.7%
– Graduate	39	18.8%
– Did not report	6	2.9%

### Survey instrument

There is a lack of standardized assessment for concussion knowledge. The literature indicates that researchers developed several surveys to assess specific aspects of concussion knowledge [8,36–38]. These studies developed surveys to examine high school athletes' knowledge of concussion symptoms, multiple concussions, and general knowledge [8,36]; symptoms, outcomes, and educational sources of concussion among high school football players [37]; correct identification of ways to get, prevent, symptoms of, and information sources of concussions with adults 18 years or older on the Porter Novelli's 2017 SummerStyle survey [38]. The survey developed for this study aimed at assessing occupational performance limitations, knowledge about concussions, and sources of concussion knowledge (Appendix 1).

Researchers explored postsecondary students' current knowledge and education regarding concussions through an anonymous online survey. The survey took approximately ten minutes to complete (based on piloting the survey with West Virginia University occupational therapy students). The first section of the instrument included demographic questions of age, sex, ethnicity, class designation, and personal history with a concussion. The participants self-reported their knowledge regarding concussion on a 5-point Likert scale from “no knowledge” to “high knowledge.” Then the participants were asked to identify their sources of concussion knowledge from a list that included a variety of campus professionals, health professionals, peers and parents, and media sources. The survey presented participants with a list of accurate and inaccurate affirmative statements regarding concussion to assess the accuracy of their knowledge, for example, “you must lose consciousness to have a concussion” or “concussion is a brain injury.” The participants indicated “yes,” “no,” or “not sure” for each statement. Participants reviewed a list of potential symptoms and identified which could occur following a concussion. In the final question, the participants identified preferred methods of future education from a list.

### Data analysis

To analyze the data, researchers utilized the statistical software SAS JMP Pro version 13 (SAS Institute Inc., NC, USA). The scoring on the ‘Yes’, ‘No’, or ‘Not Sure’ concussion knowledge survey was calculated based on either correct or incorrect; ‘Not Sure’ was scored as incorrect. The sum of correct answers was then used for analysis. Means values and standard deviations were used for the continuous variables of self-assessment of concussion knowledge and concussion knowledge survey score. Frequencies and percentages were calculated for the categorical variables of sex, ethnicity, class designation, sources of knowledge, and general concussion knowledge. Continuous variables were compared using Wilcoxon Ranks-Sum Test and *t*-test with the alpha value set at 0.05. Categorical variables were compared using Chi-square and Fisher's Exact Test with an alpha value set at 0.01 to decrease the likelihood of Type I Error.

## Results

### Sex differences

Students rated their knowledge of concussions on a 5-point Likert Scale between 1 = no knowledge and 5 = high knowledge. The median ranking for males (n = 50) was 4, and for females (n = 116), it was 3. A Wilcoxon Ranks-Sum Test was used due to the scores being non-parametric. Results indicated no significant differences in sex rating of self-knowledge Z = 0.528, p = 0.596. This suggests no difference between males and females in how they rated their knowledge of concussions.

On the concussion knowledge assessment, the mean score for all participants was 7.234 (SD = 1.45). There was a significant difference in male and female scores, t(108.030) = 2.621, p = 0.010. Females scored higher (n = 124, M = 7.435, SD = 1.398) than males (n = 59, M = 6.831, SD = 1.487). An independent sample t-test was selected because it is the most appropriate technique for analyzing the mean comparison of two independent groups. The results indicated that females performed better than males on the concussion knowledge assessment. A Chi-square and Fisher's Exact Test 2-tail test was performed for each of the questions on the concussion knowledge assessment to look at sex differences (Table 2). The chi-square was selected to test whether the categorical variables are related to each other with the Fisher exact test used when a cell had a categorical value was less than 5. The analysis compares the correct or incorrect answers by males and females. A statistically significant relationship existed between sex and knowledge of concussion of ‘must lose consciousness’ (χ^2^ [1, n = 183] = 10.830, p = 0.001) and ‘similar experiences with gender’ (χ^2^ [1, n = 183] = 10.830, p = 0.001) with females identifying the correct answer more often. The results indicate that females statistically identified the correct response for ‘must lose consciousness’ and ‘similar experiences with gender’ more than males.

**Table 2.** Chi-square and Fisher's Exact Test 2-tail significance of participants' accurate understanding of concussion by sex.

Variable	Correct		Incorrect		χ^2^	df	n	p-value
	Male	Female	Male	Female				
Concussion is a brain injury^‡^	94.92%	94.31%	5.08%	5.69%	–	–	182	1.000
Must lose consciousness	79.66%	95.16%	20.34%	4.84%	10.83	1	183	0.001^†^
Females and males have similar experiences	16.95%	42.75%	83.05%	57.26%	11.781	1	183	0.001^†^
Must get hit in the head	66.10%	76.61%	33.90%	23.39%	2.253	1	183	0.133
Can have long term consequences (>1 year)	89.83%	90.32%	10.17%	9.68%	0.011	1	183	0.917
Can affect academic performance^‡^	98.31%	95.97%	1.69%	4.03%	–	–	183	0.666
One concussion increases risk for another	89.83%	86.29%	10.17%	13.71%	0.456	1	183	0.500
Recovery time longer after second concussion^‡^	1.69%	3.25%	98.31%	96.75%	–	–	182	1.000
Chronic traumatic encephalopathy only experienced by athletes	74.58%	77.24%	25.42%	22.76%	0.156	1	182	0.693
Secondary impact syndrome occurs if you hit your head before recovering from 1st concussion	71.19%	83.06%	28.81%	16.94%	3.428	1	183	0.064

† Statistically significant at p < 0.01.

‡ Fisher's exact test two-tail significance.

### Concussion knowledge sources

Table 3 provides the Chi-square and Fisher's Exact Test analysis of where participants learned about concussions, concussion management, treatment, etc. The chi-square was selected to test whether the categorical variables are related to each other with the Fisher exact test used when a cell had a categorical value was less than 5. Examining the percentages of responses, the top 3 ways of learning about concussions, concussion management, treatment, etc., were through ‘health educator’ (44%, n = 79), ‘internet/social media’ (43%, n = 77), and ‘parents’ (29%, n = 53). Of the students who completed this survey, 56% of males (n = 32) indicated that they learned about concussions from the ‘internet and social media’ compared to 37% of females (n = 45), 35% of females (n = 42) indicated ‘parents’ compared to males at 19% (n = 11), and 46% of males (n = 26) indicated ‘television’ compared to 16% of females (n = 20). There were statistically significant differences between sex one the variables: ‘television’ [χ^2^ (1, n = 180) = 17.641, p = 0.001] and trends with ‘internet/social media’ [χ^2^ (1, n = 180) = 6.085, p = 0.014], and ‘parents’ [χ^2^ (1, n = 180) = 4.133, p = 0.042]. The results indicated that males statistically identified ‘television’ more often than females and that a possible statistical difference is trending with ‘internet/social media,’ with males selecting this option more often than females and females selecting ‘parents’ more than males for where participants learned about concussions, concussion management, treatment, etc.

**Table 3.** By sex (male = 57, female = 123), chi-square analysis and Fisher's Exact Test of where participants learned about concussions, concussion management, treatment, etc.

Variable	Agreement		Disagreement		χ^2^	df	n	p-value
	Male	Female	Male	Female				
Campus peer educator	10.53%	5.59%	89.47%	91.80%	0.259	1	179	0.611
Campus news article^‡^	7.02%	1.16%	92.98%	98.37%	–	–	–	0.081
Faculty/coursework	29.82%	20.33%	70.18%	79.67%	1.965	1	180	0.161
Friends	21.05%	22.76%	78.95%	77.24%	0.066	1	180	0.797
Health center	28.07%	27.64%	71.93%	72.36%	0.004	1	180	0.953
Health educator	40.35%	45.53%	59.65%	54.47%	0.424	1	180	0.515
Internet/social media	56.14%	36.59%	43.86%	63.41%	6.085	1	180	0.014
Leaflets, pamphlets, flyers	7.02%	8.13%	92.98%	91.87%	–	–	–	1.000
Magazines	8.77%	4.07%	91.23%	95.93%	1.645	1	180	0.200
Parents	19.30%	35.15%	80.70%	65.85%	4.133	1	180	0.042
Religion	0.00%	0.00%	100.00%	100.00%	–	–	180	n/a
Resident assistants/advisors^‡^	1.75%	0.81%	98.25%	99.19%	–	–	–	0.534
Television	45.61%	16.26%	54.39%	83.74%	17.641	1	180	0.001^†^
Other	19.30%	19.51%	80.70%	80.49%	0.001	1	180	0.973
Have not learned^‡^	3.51%	3.25%	96.49%	96.75%	–	–	–	1.000

† Statistically significant at p < 0.01.

‡ Fisher's exact test two-tail significance.

Participants received a follow-up question about where they want to learn about concussions, concussion management, treatment, etc., in the future. Students rated their top 3 sources for where they wanted to learn about concussions, concussion management, treatment, etc., in the future as health educators (56%, n = 103), health centers (54%, n = 99), and campus peer educators (36%, n = 66). Of the students who completed this survey, 23% of males (n = 14) indicated that they wanted to learn about concussions from ‘television’ compared to 10% of females (n = 12), 22% of males (n = 11) indicated ‘friends’ compared to 9% of females (n = 9%), and 7% of males (n = 4) indicated ‘religion’ compared to 2% of females (n = 2). Analysis of where participants wanted to learn about concussions, concussion management, treatment, etc., in the future, revealed trending differences between sex on ‘television’ [χ^2^ (1, n = 181) = 6.240, p = 0.013] and trends with ‘friends’ [χ^2^ (1, n = 181) = 3.452, p = 0.063] and ‘religion’ (Fisher's Exact Test p = 0.089). The results indicated that a possible statistical difference is trending with ‘television’ and ‘religion,’ with males selecting these options more than females and females selecting ‘friends’ more than males.

## Discussion

This research hypothesized that sex differences would emerge with reporting knowledge of concussions. This study found sex differences in how participants learned about concussions, where they wanted to learn about concussions in the future, and their concussion knowledge. This study adds to the literature on college students' knowledge of concussions and promotes the need for better health education.

### Sex differences

Like Wallace and colleagues' study, sex differences emerged in the concussion knowledge assessment, with females identifying more correct answers than males [22]. According to Sullivan and Molcho, differences may result from differences in the sporting environment. Males are often encouraged to play through the pain, and females are more concerned with health [8]. Females being more concerned with their health, may seek more information about their health. When paired with the difference in knowledge sources (Table 3), researchers can look at implementing interventions that target what most males identify as sources of concussion knowledge. Additional research is needed to determine which specific television, internet, and social media sources provide accurate and retainable concussion information for males as a more targeted intervention.

Parents' communication about the importance of reporting concussion symptoms influences a child's attitude about reporting and may be related to the child's perceived threat; however, there are no specific studies on sex differences in what parents tell their children about concussions [39]. Females reported that they learned about concussions more from their parents than males, which may indicate that parents communicate more with a female about concussions than a male. It is unclear why parents provide females with more concussion information; however, this may be due to parenting style. While there is currently no literature on parenting style impact on a child's sex regarding concussion knowledge, education, and reporting; however, we can look at parenting styles about childhood injury risk for some inferences. Parenting style is one factor associated with childhood injury risk [40]. Caregivers who rate themselves as more protective, concerned about safety, believe they can keep their child safe, and think they are in control of their children's health have children with fewer injuries and engage in less risk-taking behavior [41]. Those with a permissive parenting style had children with an increased risk of medically-attend injuries, possibly due to having fewer rules [42]. Kroshus and colleagues found that parents' gender differed in perceived harm associated with concussion; mothers thought having a concussion would result in more long-term health impacts than fathers [39]. However, this study did not examine whether this perception changed based on their child's sex. While this research demonstrates that parenting styles and perceived harm can influence childhood injury risk, further research is needed about sex differences in how parents approach concussion education with their children.

### Concussion knowledge sources

There is limited information on concussion knowledge sources and outcomes. Efforts at preventing initial and following concussions require knowledge of symptom identification, treatment, and what to do when concussed [14]. This study identified the top choice among participants for current and future knowledge as health educators. While it was the highest, it was still a low overall percentage. Interventions and education efforts may want to provide more tools for health educators to support college students. The expectation was for the internet/social media to be a highly-rated source of knowledge. This source can be good, but often there is a lot of misinformation, and challenging to determine if students are receiving quality concussion education. There is a need for discretion when using the internet to ensure accurate information.

The following sources were expected to be rated higher: parents and television. There is an expectation that parents of children who play sports would know about concussions due to the requirements that parents receive concussion education before the child plays a sport. According to the CDC, 54.1% of children aged 6–17 participated in a sport in the last 12 months of 2020 [43]. Cournoyer and Tripp's (2014) study of concussion knowledge in high school football players found that 54% of participants identified receiving concussion education from their parents [41]. In this study, only 30% of participants identified their parents as a source. The results of this study may be reporting a lower percentage of concussion education from parents because participants may not be athletes. Therefore, parents would not be exposed to concussion consent forms or feel the need to discuss the topic because they do not foresee a risk. The CDC (2019) worked with the National Football League and the NCAA to create nationally televised public service announcements that aired on all major television networks. In addition to this CDC broad effort, one would expect television to be higher due to news, sports, and other programs discussing the topic [43]. Still, participant selection of television was low.

Concussion education is critical to identifying and managing symptoms [14,44]. Most educational programs focus on identifying concussion symptoms for athletes; however, education must focus on more than just the student-athletes. Educating all students about the importance of disclosing and identifying symptoms is crucial to receive proper care and recovery for everyone [14,44]. Research indicates that early education intervention is essential for health and safety [45]. Interventions should be flexible to meet the needs of all college students.

CDC or expert panel materials are effective educational programs that improve concussion knowledge and attitude [3]. Kroshus and Chrisman recommend that education is through safe and supportive communication between those at risk and other stakeholders [30]. They conceptualized that education conducted within similar settings to where a person might get a concussion encourages safe and open communication between stakeholders [30]. This communication promotes well-being through multiple short messages and reminders that include diverse communities (e.g., athletes, trainers, and coaches.) to be involved. It provides real-world experience for parties to learn. According to Clark and Stanfill, education should focus on symptom recognition and the risks of continuing activities. Education is comprehensive of age, sex, family socioeconomic status, geographical location, high-risk target groups, collaborative efforts among groups involved, parental involvement, and making medical professionals accessible [45].

Based on previous studies, the recommendation for providing college student education would include the instruction as part of initial student orientation [30,44]. Based on the findings of this study, universities would use sources of knowledge like social media and television to provide educational materials. Places like recreational centers, sports fields, libraries, and parks would provide pamphlets, videos, and posters that offer short messages and reminders about the risk, concussion identification, address misconceptions, and how to receive treatment. Ideally, multiple stakeholders will promote concussion awareness and well-being at the college. With parents being another highly rated source of concussion knowledge, they would also receive information about concussions as key stakeholders. Additional stakeholders include health center workers, nurses, health practitioners, academic services, counselors, and resident assistants. This group of stakeholders can provide education and help the students post-concussion with the services they might need. Overall, education needs to meet the students where they are and provide the necessary resources.

### Limitations & future studies

The study had several limitations with methodology, interpretations, and findings. First, the study attempted to include participants from 500 universities across the United States through social networking sites and student life departments; however, participants were not required to share which social networking site they received a message from their university's student life department or what university they attended. While this effort was to keep participants anonymous, it did not enable us to gather any descriptive data on recruitment methods, different universities, part of the country responses, or why only 208 students responded out of the 500 universities contacted. Lacking this and other demographical data limits the generalizability of the findings. Future studies may want to include questions on where students were referred to the research and what university they attend to determine if any patterns exist based on referral methods and dynamics of the university education system. Second, the sample was limited to college students, and participants were predominately Caucasian (84.1%), which is higher than what is reported by the NSCRC of 47%; therefore, the results may only apply to this population [35]. Sex was also limited to males and females for analysis; unfortunately, with only one participant identifying as a transgender male and one participant as genderqueer, no meaningful statistical analysis was possible. Future studies may need more directed recruitment efforts to ensure a more representative sample. Third, participants had 15 choices for where they learned about concussions, including a write-in option and that they have not learned about concussions. These selections were not inclusive of all possible choices. Reviewing the write-in responses, we recommend additional categories, such as athletic trainers, coaches, and medical professionals, for future studies. Fourth, there are limitations to using survey methods. The concussion knowledge survey questions asked “recognition-type” questions that provided answer choices for the respondent. These questions can lead to inflated numbers indicating that the public may know more about concussions. Efforts were made to ensure that not all answers were “Yes”; several questions were added based on misconceptions about concussions. Future studies may want to consider mixed methods with qualitative interviews to provide more immersive research into the complexities, thoughts, and misconceptions college students have about concussions.

Parents educating their children is another crucial aspect that could use further research. Parents can play a vital role in promoting concussion knowledge and awareness [34]. Females scored higher on the concussion knowledge assessment and reported learning about concussions more from parents than males. Future concussion education research needs to explore how parents are communicating concussion information. Targeting parents for concussion education intervention could lead to long-term concussion awareness as the child becomes an adult.

Results are exploratory and valuable in identifying possible concussion health education methods through which participants learned about concussions, concussion management, treatment, etc., and how they want to discover more about this in the future. Further research is needed in studying sex knowledge differences to provide a better understanding of myths, misconceptions, and current knowledge of concussions to offer a more targeted education. The NCAA currently mandates concussion education for all athletes [23]; however, this age group has the second highest rate of TBI-related emergency department visits, the second highest rate for TBI-related hospitalization, and the third highest rate for TBI-related deaths [2]. With a lack of focus on concussion education with non-athletes, future researchers should develop educational programs to reach all college students.

## Conclusion

With approximately three million concussions being recorded annually in the United States, there is a need for better education and intervention [2]. While a wide range of media is used to provide concussion education, this study provides a preliminary analysis of concussions and education for a college student population that does not explicitly target college athletes [31]. One important finding of this study was the sex differences that emerged with concussion knowledge, supporting previous research findings [10,22,24]. With females scoring higher than males, it is essential to look at sources of knowledge to identify why there is a difference. With much of the focus on concussion education being on student-athletes, there is a need to focus beyond the athlete and identify different concussion knowledge sources to aid in developing educational interventions [44]. Based on the participants in this study, the key findings and recommendations are that colleges and universities should focus their efforts on health educators, health centers, and internet/social media programs to provide concussion education. The recommendation for college non-athlete students' concussion education would be to provide information to students as they enter college as part of their orientation with a health educator, with additional information accessible at other locations across campus through their health centers and internet/social media programs. Education must teach college students, family, friends, and others. Education on signs and symptoms, attitude toward concussion, intention to report, and behavior changes can reduce concussion rates [3]. Education on college campuses will help increase awareness, prevention, and treatment to improve quality of life.

**Summary points**

- Traumatic brain injuries are a critical public health issue.
- There is a need for colleges/universities to provide concussion education to all students, athletes, and non-athletes.
- College student concussion education is essential to concussion identification, treatment, reporting, and recovery.
- It is essential for peers, family, colleagues, and bystanders to know concussion signs and symptoms; concussed individuals may be unable to identify the symptoms in themself.
- A lack of knowledge and misconceptions about concussions can lead to further harmful actions and stop people from seeking medical treatment.
- A total of 208 postsecondary education students participated in a survey on their current knowledge and education regarding concussions.
- Sex differences emerged in the concussion knowledge assessment, with females identifying more correct answers than males.
- Students identified their top choices for where they wanted to learn about concussions as health educators, health centers, and campus peer educators.
- It is essential to look at sources of knowledge to identify where to target educational interventions on college campuses will help increase awareness, prevention, and treatment to improve quality of life.
